# Air pollution is associated with abnormal left ventricular diastolic function: a nationwide population-based study

**DOI:** 10.1186/s12889-023-16416-x

**Published:** 2023-08-12

**Authors:** Congyi Zheng, Haosu Tang, Xin Wang, Zuo Chen, Linfeng Zhang, Jiayin Cai, Xue Cao, Runqing Gu, Yixin Tian, Zhen Hu, Gang Huang, Zengwu Wang

**Affiliations:** 1 Division of Prevention and Community Health, National Center for Cardiovascular Disease, National Clinical Research Center of Cardiovascular Disease, State Key Laboratory of Cardiovascular Disease, Fuwai Hospital, Peking Union Medical College & Chinese Academy of Medical Sciences, No. 15 (Lin), Fengcunxili, Mentougou District, Beijing, 102308 China; 2 State Key Laboratory of Numerical Modeling for Atmospheric Sciences and Geophysical Fluid Dynamics, Institute of Atmospheric Physics, Chinese Academy of Sciences, Beijing, 100029 China; 3 University of Chinese Academy of Sciences, Beijing, 100049 China

**Keywords:** Air pollution, Particulate matter, Cardiac dysfunction, Risk factor, Population

## Abstract

**Background:**

Air pollution is a growing public health concern of global significance. Till date, few studies have explored the associations between air pollutants and cardiac imaging phenotypes. In this study, we aim to explore the association of ambient air pollution and abnormal left ventricular diastolic function (ALVDF) among a large-scale free-living population.

**Methods:**

The participants were from a national representative large-scale cross-sectional study, i.e., the China Hypertension Survey (CHS), 2012–15. After exclusion, 25,983 participants from 14 provinces and 30 districts in China were included for the final analysis. The annual average ambient PM_2.5_, PM_10_ and NO_2_ concentrations were obtained from the chemical data assimilation system (ChemDAS). The clinical evaluation of left ventricular function was conducted in the survey field which was based on echocardiography. Grading diastolic dysfunction was based on Recommendations for the evaluation of left ventricular diastolic function by echocardiography (2009).

**Results:**

The mean age of 25,983 participants was 56.8 years, 46.5% were male, and the crude prevalence of GradeI-III ALVDF were 48.1%, 1.6% and 1.1%, respectively. The ORs (95% CI) for ALVDF in the fully adjusted model were 1.31 (1.11–1.56), 1.11 (1.01–1.21) and 1.18 (0.90–1.54) for an increase of 10 μg/m^3^ of PM_2.5_, PM_10_ and NO_2_, respectively. And for different grades of ALVDF, elevated concentration of PM_2.5_ and PM_10_ exposures significantly increased the risk of gradeIinstead of gradeII ~ III ALVDF. There was a positive linear and “J” shape concentration–response association between annual average ambient PM_2.5_ and NO_2_ and the ALVDF risk assessed by the restricted cubic spline. The exposure level of most participants to PM_10_ was less than 130 μg/m^3^, and the risk of ALVDF increased significantly with the concentration rise.

**Conclusions:**

This large-scale nationwide population study demonstrated a significantly positive association between ambient PM_2.5_, PM_10_ and NO_2_ with ALVDF, especially for mild ALVDF. The functional abnormality may partially explain the enhanced cardiovascular morbidity and mortality associated with air pollution, which highlights the importance of appropriate interventions to reduce ambient air pollution in China.

**Supplementary Information:**

The online version contains supplementary material available at 10.1186/s12889-023-16416-x.

## Introduction

Air pollution is a growing public health concern of global significance. More than 90% of the global population is exposed to PM_2.5_ levels exceeding World Health Organization air quality guidelines (AQG) of < 10 µg/m^3^ and low/middle-income countries experience the highest burden [1]. Recent studies have indicated that the global mortality and morbidity burden of cardiovascular disease (CVD) associated with air pollutants is dramatically greater than what has been thought up to now [2, 3].

Left ventricular diastolic dysfunction (LVDD), an early sign of cardiac dysfunction, is a predictor of fatal and/or nonfatal cardiovascular events [4, 5]. Even in asymptomatic patients, mild or moderate diastolic dysfunction was associated with a higher mortality risk in comparison with normal ones [6, 7]. Although air pollution has been well documented a close association with CVD morbidity and mortality [8], few studies have found a detectable adverse association between air pollutants with cardiac imaging phenotypes in asymptomatic individuals [9–11]. Our previous study has found the relationship between LVDD and CVD deteriorates in worse ambient air pollution conditions through a prospective cohort study [12]. But there has been still a paucity of information about the influence of ambient air pollution on LVDD among a large-scale free-living population from a nationwide study. Moreover, the majority of researches concerning air pollution and cardiovascular health only focuses on ambient pollutants, however, it cannot be ignored that significant evidence exists demonstrating that household air pollution such as solid fuels or secondhand smoke also has an independent deleterious effect on cardiac diastolic function or CVD event [13–15].

Thus, the current study was pooled to firstly explore the association between long-term major ambient pollutants (PM_2.5_, PM_10_ and NO_2_) exposure and abnormal left ventricular diastolic function (ALVDF) in a representative Chinese population after adjusted for household air condition and other primary risk factors. We hypothesized that annual average ambient air pollutants quantified in the survey year had a detectable adverse association with ALVDF among the asymptomatic individuals.

## Methods

The data that support the findings of this study are available from the corresponding author upon reasonable request.

### Study design and population

The data was from the China Hypertension Survey (CHS) a national representative large-scale cross-sectional study, and the detailed description of the study has been published previously [16, 17]. In brief, the CHS employed 4-stage stratified multistage random sampling method to obtain nationwide aged ≥ 35 years subjects from 14 provinces in 2012–15. The written informed consent was obtained from each participant. The Ethics Committee of Fuwai Hospital (Beijing, China) approved the study.

**Fig. 1:**
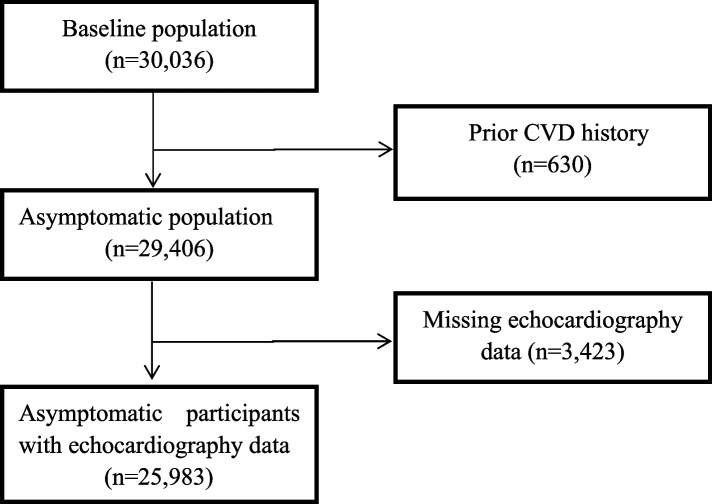
Flow diagram of participants recruitment. CVD, cardiovascular diseases

After excluding 630 participants with prior CVD history and 3,423 participants with missing echocardiography data, 25,983 participants from 14 provinces and 30 districts were included for the final analysis (Fig. 1).

### Assessment and grading left ventricular diastolic function

The clinical evaluation of left ventricular function was conducted in the survey field, which was based on echocardiography, and the collection data of cardiac ultrasound examination in the questionnaire included M-mode and two-dimensional measurements, heart value structure, Doppler flow parameters. All experienced echocardiographers were trained using the protocol. And the difficult-to-diagnose special cases were discussed with the experts from the coordination center.

Grading diastolic dysfunction was based on Recommendations for the evaluation of left ventricular diastolic function by echocardiography (2009) [6]. The grading scheme is grade I (impaired relaxation pattern), grade II (pseudo normal), and severe (restrictive filling) or grade III. ALVDF group included grade I- III.

### Ambient air pollution exposure assessment

**Fig. 2:**
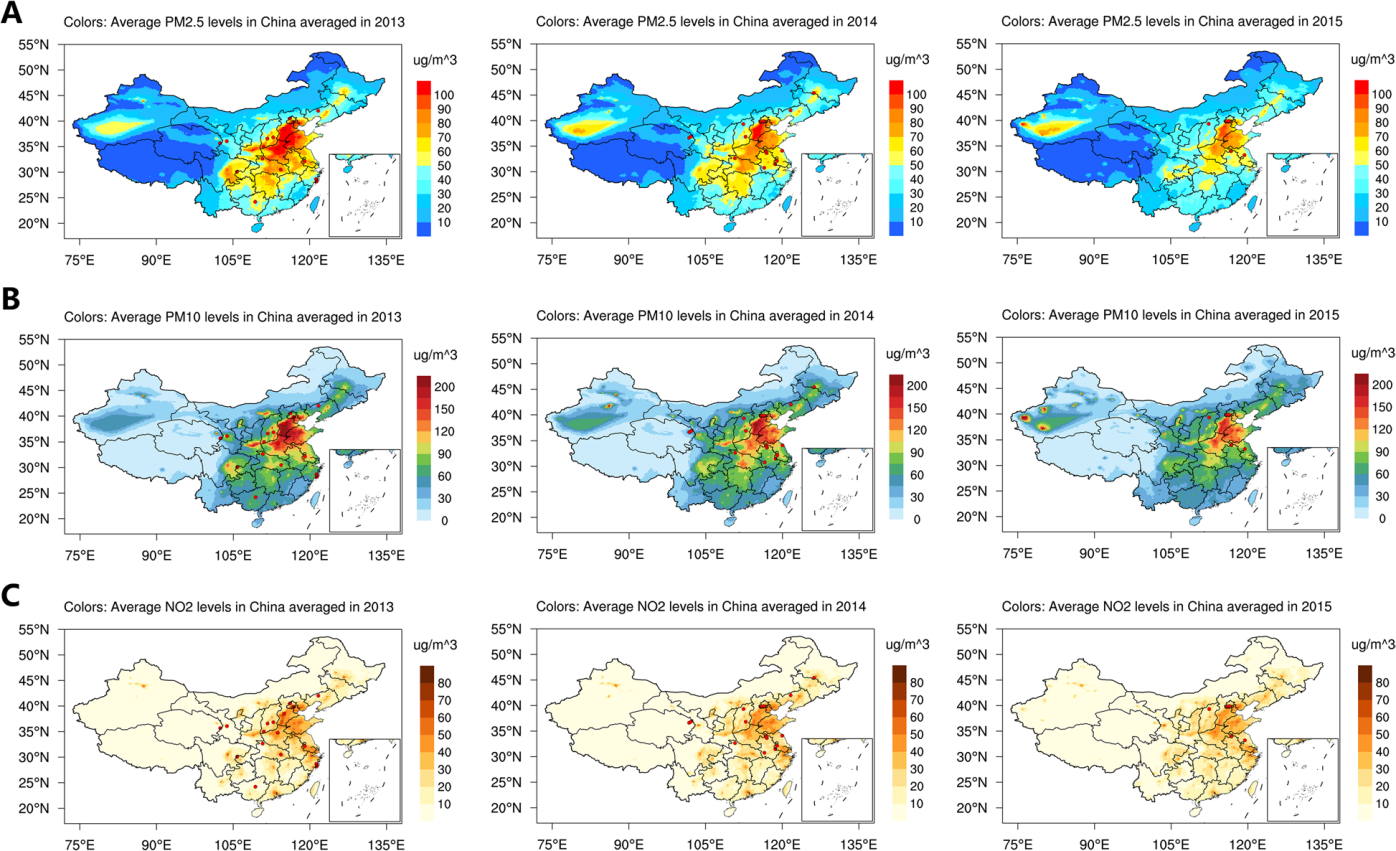
Average PM_2.5_ (**A**), PM_10_ (**B**) and NO_2_ (**C**) levels in China, 2013–2015. The red dots are the baseline survey sites of this study in each year

The annual average PM_2.5_, PM_10_ and NO_2_ concentrations were obtained from a high-resolution air quality reanalysis dataset over China from 2013 to 2018 [18] which was produced by the chemical data assimilation system (ChemDAS) developed by the Institute of Atmospheric Physics, Chinese Academy of Sciences (Fig. 2). This dataset has high spatial (15 km) and temporal (1 h) resolutions, the qualities of which have been assessed by the independent observations and demonstrate generally good *R*^*2*^ values of 0.74–0.86 [18]. The above air quality reanalysis data were interpolated to each subject of the current study according to his/her residential address via the bilinear interpolation method [19]. In the present study, we explored the effects of air pollutant annual average concentrations of the survey year for each participant except for 317 participants who were registered in December 2012, and their exposure data were estimated by the annual average concentrations of 2013.

### Covariates

At CHS, the trained workers administered a standardized electronic questionnaire to collect information on demographic characteristics (age, sex, ethnicity, area and education level), lifestyle behaviors (smoking, passive smoking and alcohol consumption), family CVD history and medical history. Altitude of each survey sites were estimated from ~ 2,419 homogenized surface meteorological observation stations [20]. Blood pressure was measured with the OMRON HBP-1300 Professional Portable Blood Pressure Monitor (OMRON, Kyoto, Japan) three times, and the average of the three readings was used for analysis. Body weight was obtained using OMRON body fat and weight measurement device (V-body HBF-371; Omron, Kyoto, Japan). Laboratory analyses were performed by a central core laboratory (Beijing Adicon Clinical Laboratories, INC, Beijing, China) using standardized techniques. All blood samples were obtained in the morning after at least 8 h overnight fast.

Each participant was asked to provide detailed information about indoor air pollution exposure related to solid heating fuels, indoor ventilation, cookstove ventilation and passive smoke. Participants who used heating in winter were asked additional questions about the primary fuel type used, which included central heating (a system generating heat in a centralized location distant from residential areas and distributing the heat to individual households via underground hot water or steam pipes), gas, coal, wood/charcoal, crop straw and other unspecified fuels. Coal, wood /charcoal, crop straw were considered “solid fuels,” while gas and central heating were considered “clean fuels” because they tended to generate much less air pollution than solid fuels [21]. Nonsmokers were asked “Are you exposed to second-hand smoke usually?”, “How many days per week are you exposed to second-hand smoke usually?” Those who answered “none” were categorized as non-exposed to second-hand smoke and all others were categorized as exposed to second-hand smoke.

### Statistical analysis

The characteristics of the study population were described by gender, using numbers with the corresponding percentages for categorical variables and means with the standard deviation for continuous variables, group differences were assessed by *χ*^2^ test, respectively. Odds ratios (ORs) (95% CI) for the associations of air pollution with LV diastolic function (abnormal vs. normal) or grade I-III LVDD were calculated using multivariate two-level logistic regression analysis (All intraclass correlation coefficients were > 20% in all adjusted models) that included a random cluster effect (district), and the interaction term was added to estimate the effect on LVDD in stratified analysis by sex, age, smoking and BMI. Fully-adjusted multivariable models included the following covariates: age, sex, areas (urban, rural), habitation altitude (< 1500 m, 1500-3500 m, ≥ 3500 m), ethnicity (Han, minority), education (primary, middle, high), smoke (current, former, never), drinking, family history of CVD, obesity (normal, overweight, obesity), hypertension, hyperlipidemia, diabetes, medical therapy (anti-hypertensive, hypoglycaemic drug and statin), solid heating fuel use, frequent indoor ventilation and secondhand smoke. Furthermore, we used restricted cubic spline regression fitted for multivariate logistic models with 3 knots (PM_2.5_: 50, 70 and 90 μg/m^3^; PM_10_: 100, 130 and 150 μg/m^3^; NO_2_: 35, 45 and 55 μg/m^3^) to examine the concentration–response association between long-term exposure to the three different ambient air pollutants and ALVDF, respectively. The optimal cutoff values of the curves were selected according to the distribution of the air pollutants exposure concentration (Fig. S1).

All the analyses were carried out using SAS version 9.3 (SAS institute, Cary, NC, USA) and the maps were constructed with the NCAR Command Language (http://www.ncl.ucar.edu). The two-sided *P* values < 0.05 were considered statistically significant.

## Results

### Baseline characteristics of the study population

There were a total of 25,983 participants considered for this study, and their baseline demographic characteristics of the participants were presented in Table 1. At baseline, the mean age of participants was 56.8 years, 46.5% were male, the crude prevalence of ALVDF was 50.8%, and GradeI-III ALVDF were 48.1%, 1.6% and 1.1%, respectively. The annual average ambient PM_2.5_, PM_10_ and NO_2_ concentration of the survey year were 62.77, 94.22 and 29.87 μg/m^3^. 24.0% participants were reported using solid fuel and 8.5% of them exposed to the secondhand smoke. And male subjects tended to have higher blood pressure, triglycerides, fasting plasma glucose, higher prevalence of ALVDF and lower ambient PM_2.5_ as well as indoor air pollution exposure level.

**Table 1:** Baseline characteristics of the study participants

	Total (*n* = 25,983)	Male (*n* = 12,080)	Female (*n* = 13,903)	*P*
**Demographics**				
Age (years)	56.81 ± 13.20	57.55 ± 13.33	56.17 ± 13.06	< 0.001
Rural (%)	13,816 (53.2)	6,318 (52.3)	7,498 (53.9)	0.009
Habitation altitude	439.30 ± 687.07	430.72 ± 684.37	446.76 ± 689.35	0.061
Education (≥ Middle school)	12,784(49.2)	6,883(57.0)	5,901(42.4)	< 0.001
**Clinical characteristics**				
Smoking (%)				
Current	6,443(24.8)	5,964(49.4)	479(3.4)	< 0.001
Former	1,586(6.1)	1,467(12.1)	119(0.9)	
Never	17,954(69.1)	4,649(38.5)	13,305(95.7)	
Alcohol drinking (%)	7,159(27.6)	6,210(51.4)	949(6.8)	< 0.001
Family history of CVD (%)	3,150 (12.1)	1,364(11.3)	1,786(12.8)	< 0.001
SBP (mmHg)	132.62 ± 20.42	133.37 ± 19.40	131.97 ± 21.24	< 0.001
DBP (mmHg)	77.25 ± 11.10	79.28 ± 11.17	75.49 ± 10.73	< 0.001
BMI (Kg/m^2^)	24.62 ± 3.50	24.42 ± 3.37	24.79 ± 3.60	< 0.001
Total cholesterol (mmol/L)	4.81 ± 0.99	4.71 ± 0.96	4.89 ± 1.01	< 0.001
HDL-cholesterol (mmol/L)	1.37 ± 0.35	1.32 ± 0.35	1.40 ± 0.34	< 0.001
LDL-cholesterol (mmol/L)	2.82 ± 0.82	2.75 ± 0.80	2.87 ± 0.84	< 0.001
Triglycerides (mmol/L)	1.46 ± 1.03	1.50 ± 1.13	1.43 ± 0.93	< 0.001
FPG (mmol/L)	5.65 ± 1.59	5.71 ± 1.64	5.60 ± 1.54	< 0.001
Medical therapy (%)				
Anti-hypertensive drug	5,246(20.2)	2,366(19.6)	2,880(20.7)	0.024
Hypoglycaemic drug	1310(5.0)	603(5.0)	707(5.1)	0.73
Statin	973(3.7)	428(3.5)	545(3.9)	0.11
LVDD (%)				
Grade I	12,491(48.1)	6,133(50.8)	6,358(45.7)	< 0.001
Grade II	407(1.6)	214(1.8)	193(1.4)	
Grade III	289(1.1)	149(1.2)	140(1.0)	
**Baseline annual average ambient pollutants**				
PM_2.5_ (μg/m^3^)	62.77 ± 22.92	62.44 ± 22.70	63.06 ± 23.11	0.029
PM_10_ (μg/m^3^)	94.22 ± 40.60	94.01 ± 40.24	94.40 ± 40.91	0.43
NO_2_ (μg/m^3^)	29.87 ± 13.27	29.91 ± 13.15	29.84 ± 13.38	0.67
**Indoor Pollutants and related confounding factors**				
Solid heating fuels use (%)	6,228(24.0)	2,833(23.5)	3,395(24.4)	0.068
Secondhand smoke (%)	2,218(8.5)	495(4.1)	1,723(12.4)	< 0.001
Indoor ventilation frequently (%)	13,191(50.8)	6,092(50.4)	7,099(51.1)	0.31
Cookstove ventilation use (%)	15,312(85.5)	7,093(85.0)	8,219(85.9)	0.090

Numbers are mean ± SD or no.(%). BMI, indicates body mass index

*CVD* Cardiovascular disease, *SBP* Systolic blood pressure, *DBP* Diastolic blood pressure, *FPG* Fasting plasma glucose, *HDL-cholesterol* High density lipoprotein cholesterol, *LDL-cholesterol* Low-density lipoprotein cholesterol, *LVDD* Left ventricular diastolic dysfunction, included impaired relaxation pattern (Grade I), pseudo normal (Grade II), and restrictive filling (Grade III)

### Air pollution and ALVDF

Crude and multivariate-adjusted ORs and 95% CI for associations of ALVDF with ambient air pollution were presented in Table 2. In the crude model, higher PM_2.5_, PM_10_ and NO_2_ exposures were significantly associated with the increased risks of ALVDF. The ORs (95% CI) for ALVDF in the fully adjusted model (Model 4) were 1.31 (1.11–1.56), 1.11 (1.01–1.21) and 1.18 (0.90–1.54) for per 10 μg/m^3^ increment of PM_2.5_, PM_10_ and NO_2_, respectively.

**Table 2:** Adjusted odds ratio (95% CI) of ALVDF associated with ambient air population

	**Per 10 μg/m**^**3**^** increment**		
	**PM**_**2.5**_	**PM**_**10**_	**NO**_**2**_
Crude model	2.04 (1.70–2.44)	1.53 (1.39–1.68)	2.40 (1.87–3.01)
Adjusted Model 1^a^	1.54 (1.31–1.82)	1.21 (1.11–1.32)	1.44 (1.15–1.81)
Adjusted Model 2^b^	1.54 (1.30–1.84)	1.20 (1.10–1.31)	1.41 (1.09–1.82)
Adjusted Model 3^c^	1.49 (1.25–1.78)	1.16 (1.06–1.27)	1.26 (0.97–1.63)
Adjusted Model 4^d^	1.31 (1.11–1.56)	1.11 (1.01–1.21)	1.18 (0.90–1.54)

*ALVDF* abnormal left ventricular diastolic function

^a^Model 1: Crude model + adjusted for age and sex

^b^Model 2: Model 1 + adjusted for areas, habitation altitude, ethnicity and education

^c^Model 3: Model 2 + adjusted for smoke, drinking, family history of CVD, obesity, hypertension, hyperlipidemia, diabetes, medical therapy

^d^Model 4: Model 3 + adjusted for solid heating fuels, passive smoke, indoor ventilation and cookstove ventilation

The full-adjusted ORs (95% CI) of grade I ALVDF associated with per 10 μg/m^3^ increment of ambient PM_2.5_, PM_10_ and NO_2_ were 1.42 (1.18–1.70), 1.15 (1.04–1.26) and 1.28 (0.97–1.68), respectively (Table 3). And there was no significant association between the three pollutants exposure and the risk of grade II or III ALVDF. The full-adjusted ORs (95% CI) of grade II ALVDF related with per 10 μg/m^3^ increment of ambient PM_2.5_, PM_10_ and NO_2_ were 0.85 (0.60–1.19), 0.88 (0.72–1.07) and 0.80 (0.41–1.58), respectively. And the full-adjusted ORs (95% CI) of grade III ALVDF related with per 10 μg/m^3^ increment of ambient PM_2.5_, PM_10_ and NO_2_ were 0.97 (0.47–1.99), 0.97 (0.66–1.43) and 0.22 (0.04–1.30), respectively (Table 3).

**Table 3:** Full-adjusted odds ratio (95% CI) of different grade of ALVDF associated with ambient air population

ALVDF grade	Per 10 μg/m^3^ increment		
	**PM**_**2.5**_	**PM**_**10**_	**NO**_**2**_
I	1.42 (1.18–1.70)	1.15 (1.04–1.26)	1.28 (0.97–1.68)
II	0.85 (0.60–1.19)	0.88 (0.72–1.07)	0.80 (0.41–1.58)
III	0.97 (0.47–1.99)	0.97 (0.66–1.43)	0.22 (0.04–1.30)

All models were adjusted age, sex, areas, habitation altitude, ethnicity, education, smoke, drinking, family history of CVD, obesity, hypertension, hyperlipidemia, diabetes, medical therapy, solid heating fuels, passive smoke, indoor ventilation and cookstove ventilation

*ALVDF* Abnormal left ventricular diastolic function

### Concentration–response between air pollution and ALVDF risk

**Fig. 3:**
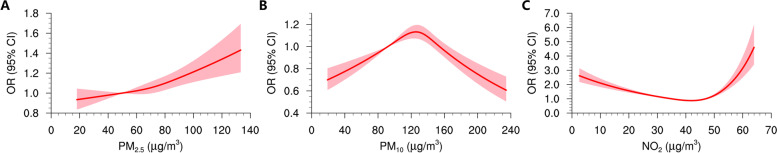
Concentration–response association between annual average ambient PM_2.5_ (**A**), PM_10_ (**B**) and NO_2_ (**C**) with the risk of ALVDF. Restricted cubic spline regression was used to fit for multivariate logistic models with 3 knots (PM_2.5_: 50, 70 and 90 μg/m^3^; PM_10_: 100,130 and 150 μg/m^3^; NO_2_: 35, 45 and 55 μg/m^3^). The red curves represent estimates of odds ratios, and the light red shading areas represent pointwise 95%CI. All estimates were adjusted for age, sex, areas, habitation altitude, ethnicity, education, smoke, drinking, family history of CVD, obesity, hypertension, hyperlipidemia, diabetes, medical therapy, solid heating fuels, passive smoke, indoor ventilation and cookstove ventilation. ALVDF, abnormal left ventricular diastolic function

In Fig. 3, we estimated that there was a positive linear concentration–response association between annual average ambient PM_2.5_ with the risk of ALVDF risk (Overall *P* < 0.001; Non_linear *P* = 0.29); The plot of NO_2_ (“J” shape) showed a substantial reduction of the ALVDF risk in low level group, which reached the lowest risk around 45 μg/m^3^, there after the risk increased, and the increasing trend was generally rapid (Overall *P* < 0.001; Non_linear* P* < 0.001); The exposure level of the most participants to PM_10_ was less than 130 μg/m^3^, which compromised > 90% of the sample size, and the risk of ALVDF increased significantly with the concentration rise in this interval (Overall *P* < 0.001; Non_linear* P* < 0.001).

## Discussion

To our knowledge, this is the first study evaluating the effect of ambient air pollution on ALVDF among a large-scale asymptomatic population. We found that there was a positive linear and “J” shape concentration–response association between annual average ambient PM_2.5_ and NO_2_ with the ALVDF risk assessed via the restricted cubic spline.

Consistent with the current study, air pollution especially PM_2.5_ and/or NO_2_ has been well demonstrated a close association with heart failure incidence and heart failure mortality in a systematic review and meta-analysis [8]. And in a recent population-based study of 5.1 million Canadian, Bai et al. found that the ORs of congestive heart failure corresponding to each interquartile range increase in exposure were 1.05 (95% CI: 1.04–1.05) for PM_2.5_ and 1.02 (95% CI: 1.01–1.04) for NO_2_ [22]. And a few studies have evaluated the associations between household secondhand smoke and solid fuels heating with heart failure or heart failure mortality [12, 23]. The previously published analysis of the UK Biobank Population Imaging Study of 3,920 asymptomatic subjects showed that higher past exposure to ambient PM_2.5_ and NO_2_ was associated with cardiac ventricular dilatation, a marker of adverse remodeling that often precedes heart failure development which was consistent with our results [9]. In accordance, Jafar and his colleagues investigated the association of exposure to occupational air pollution and cardiac function in the workers of the steel industry, 50 male workers of the agglomeration and coke-making parts of the Esfahan Steel Company were randomly selected and 50 workers in the administrative parts were studied as controls. They found that left ventricular ejection fraction was lower in workers of the agglomeration/coke-making parts than in controls (mean difference = 5 to 5.5%, *P* < 0.001) [10]. However, there was still a paucity of information in the current literatures about the ambient air pollution effect on cardiac morpho-functional phenotypes when considering the indoor air condition simultaneously among a large-scale representative population.

Consistent with the previous study [24], a significant positive effect was found when we assumed a simple linear relationship between PM_10_ and ALVDF risk. And in the current study, there was a nonlinear relationship (“ ∩ ”shape) between PM_10_ and the risk of ALVDF, assessed by the restricted cubic spline; the exposure level of most participants to PM_10_ was less than 130 μg/m^3^, and the risk of ALVDF increased significantly with the concentration rise. Similar to our study, there was also a “ ∩ ”shape association between long-term exposure to soil particles and mortality in the southeastern United States [25]. Till now, there has little investigations on the assessment the concentration–response between ambient PM_10_ and ALVDF. Therefore, the findings of the present study may provide essential evidence in this aspect.

The possible molecular mechanisms of the air pollution–mediated systemic CVD risk were: 1) endothelial barrier dysfunction/disruption; 2) inflammation, involving both innate and adaptive immune components; 3) prothrombotic pathways; 4) autonomic imbalance favoring sympathetic tone via afferent pathways the upper airways and/or lung; 5) central nervous system effects on metabolism and hypothalamic-pituitaryadrenal axis activation; and 6) epigenomic changes; and among all the pollutants PM_2.5_ is the most important environmental risk factor contributing to global cardiovascular mortality and disability [26, 27]. However, the mechanism of the air pollution–mediated ALVDF risk is still not clear.

In addition, we observed that elevated pollutant concentration exposures significantly increased the risk of grade I instead of grade II ~ III ALVDF. One of the possible explanations was that the effect of air pollution on cardiac function was relatively limited which could not be enough to result in severe ALVDF.

The current study firstly reported the effect of long-term exposures to ambient air pollutants on ALVDF. The strengths of this study include a large well-representative sample size, high accuracy estimation of ambient air pollution and comprehensive information about household air pollution, exposure concentration related factors (eg, indoor ventilation and cookstove ventilation) as well as various covariates especially the indoor air pollutions which were the potential confounding factors in the adjusted model. However, several limitations should be noted in this study. First, the evaluation of left ventricular function was only conducted in the baseline survey field. Thus, we could only explore the association between air pollution and ALVDF in baseline instead of the causal relationship. Secondly, there was a lack of nationwide PM_2.5_ observations in 2012 in China, so we could only use the annual average of the survey year rather than one-year average prior to survey, which may limit the variation of exposure levels. Additionally, we did not have data on some important covariates, such as dietary habit, which was ignored in the model. Finally, less than 2% of our free-living participants were diagnosed as moderate (Grade II) or Severe (III) LVDD, which may reduce the power of the analysis about the separate different LVDD grades.

## Conclusions

This large-scale nationwide population study demonstrated the significantly association between ambient PM_2.5_, PM_10_ and NO_2_ with ALVDF, especially for mild ALVDF. The functional abnormality may partially explain the increased cardiovascular morbidity and mortality associated with air pollution. Thus, appropriate interventions to reduce air pollution may promote great benefits to public health potentially via the decrease of ALVDF risk and the adverse events induced by cardiac dysfunction.

### Supplementary Information

**Additional file 1: Figure S1. **The distribution of the concentrations of (A) PM_2.5_, (B) PM_10_ and (C) NO_2_ in the present study. **Appendix S1.** List of the China Hypertension Survey Investigators.

## Data Availability

All data that support the findings of this study could be available from the corresponding author upon reasonable request.
