# Supplementary material for: Air pollution is associated with abnormal left ventricular diastolic function: a nationwide population-based study
Source: BMC Public Health. 2023 Aug 12;23:1537. doi: 10.1186/s12889-023-16416-x (PMC10422745; doi:10.1186/s12889-023-16416-x)
Supplement: Supplementary file 1 — Additional file 1: Figure S1. The distribution of the concentrations of (A) PM2.5, (B) PM10 and (C) NO2 in the present study. Appendix S1. List of the China Hypertension Survey Investigators. [file 12889_2023_16416_MOESM1_ESM.docx]

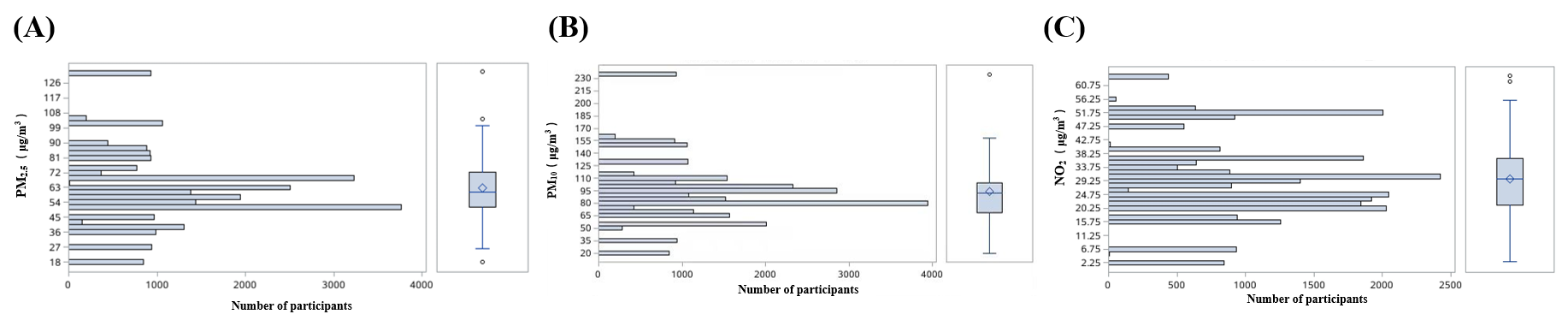


**Figure S1.** The distribution of the concentrations of (A) PM_2.5_, (B) PM_10_ and (C) NO_2_ in the present study.

**Appendix S1**

**List of the China Hypertension Survey Investigators**

This study was accomplished through the fine work of the staff at the national level. For a partial listing of colleagues see the follows (provinces sorted as alphabetical order):

**Anhui**: Liqun Hu, Hongqi Li, Qi Zhang, Guang Yan, Anhui Provincial Hospital, Hefei, Anhui, China; Fangfang Zhu, Anhui Institute of Cardiovascular Disease, Hefei, Anhui, China.

**Beijing**: Xianghua Fang, Chunxiu Wang, Shaochen Guan, Xiaoguang Wu, Hongjun Liu, Chengbei Hou, Xuanwu Hospital, Capital Medical University, Beijing, China.

**Chongqing**: Han Lei, Wei Huang, Nan Zhang, First Affiliated Hospital of Chongqing Medical University, Chongqing, China; Ge Li, Lihong Mu, Xiaojun Tang, Chongqing Medical University, Chongqing, China.

**Fujian**: Ying Han, Huajun Wang, Dongjie Lin Liangdi Xie, First Affiliated Hospital of Fujian Medical University, Fuzhou, Fujian, China; Daixi Lin, Fujian medical university, Fuzhou, Fujian, China.

**Gansu**: Jing Yu, Xiaowei Zhang, Wei Liang, Heng Yu, Qiongying Wang, Lanzhou University Second Hospital, Lanzhou, Gansu, China; Lan Yang, Maternal and Child Care Service Centre, Lanzhou, Gansu, China.

**Guangdong**: Yingqing Feng, Yuqing Huang, Guangdong General Hospital, Guangzhou, Guangdong, China; Peixi Wang, Jiaji Wang, Guangzhou Medical University, Guangzhou, Guangdong, China; Harry HX Wang, Sun Yat-Sen University, Guangzhou, Guangdong, China; Songtao Tang, Community Health Services Center of Liaobu, Dongguan, Guangdong, China.

**Guangxi**: Tangwei Liu, Rongjie Huang, Zhiyuan Jiang, Haichan Qin, First Affiliated Hospital of Guangxi Medical University, Nanning, Guangxi, China.

**Guizhou**: Guoqin Liu, Zhijun Liu, Wenbo Rao, Zhen Chen, Yalin Chu, Fang Wu, Zunyi Medical University, Zunyi, Gouzhou, China.

**Hainan**: Haitao Li, Jianlin Ma, Tao Chen, Hainan General Hospital, Haikou, Hainan, China; Ming Wu, Health and Family Planning Commission of Hainan, Haikou, Hainan, China.

**Hebei**: Jixin Sun, Yajing Cao, Yuhuan Liu, Center for Disease Prevention and Control of Hebei, Shijiazhuang, Hebei, China; Zhikun Zhang, Center for Disease Prevention and Control of Tangshan, Tangshan, Hebei, China; Yanmei Liu, Center for Disease Prevention and Control of Langfang, Langfang, Hebei, China; Dejin Dong, Center for Disease Prevention and Control of Xingtai, Xingtai, Hebei, China; Guangrong Li, Center for Disease Prevention and Control of Dingzhou, Dingzhou, Hebei, China.

**Heilongjiang**: Hong Guo, Lihang Dong, Haiyu Zhang, Fengyu Sun, Xingbo Gu, Ye Tian, First Affiliated Hospital of Harbin Medical University, Haerbin, Heilongjiang, China.

**Henan**: Kaijuan Wang, Chunhua Song, Peng Wang, Hua Ye, Zhengzhou University, Zhengzhou, Henan, China; Wei Nie, Shuying Liang, Henan Academy of Medical Sciences, Zhengzhou, Henan, China.

**Hubei**: Congxin Huang, Fang Chen, Yan Zhang, Heng Zhou, Jing Xie, Jianfang Liu, Department of Cardiology, Renmin Hospital of Wuhan University, Wuhan, Hubei, China.

**Hunan**: Hong Yuan, Chengxian Guo, Third Xiangya Hospital, Central South University, Changsha, Hunan, China; Yuelong Huang, Biyun Chen, Center for Disease Control and Prevention of Hunan, Changsha, Hunan, China.

**Inner Mongolia**: Xingsheng Zhao, Wenshuai He, Xia Wen, Yanan Lu, Inner Mongolia people's hospital, Hohhot, Inner Mongolia, China.

**Jiangsu**: Xiangqing Kong, Ming Gui, Wenhua Xu, Yan Lu, Jun Huang, First Affiliated Hospital of Nanjing Medical University, Nanjing, Jiangsu, China; Min Pan, Affiliated Hospital of Nantong University, Nanjing, Jiangsu, China; Jinyi Zhou, Ming Wu, Center for Disease Control and Prevention of Jiangsu, Nanjing, Jiangsu, China.

**Jiangxi**: Xiaoshu Cheng, Huihui Bao, Xiao Huang, Kui Hong, Juxiang Li, Ping Li, Second Affiliated Hospital of Nanchang University, Nanchang, Jiangxi, China.

**Jilin**: Bin Liu, Junduo Wu, Longbo Li, Yunpeng Yu, Yihang Liu, Chao Qi, Second Hospital of Jilin University, Changchun, Jilin, China.

**Liaoning**: Jun Na, Li Liu, Yanxia Li, Guowei Pan, Center for Disease Prevention and Control of Liaoning, Shenyang, Liaoning, China; Degang Dong, Peng Qu, Health and Family Planning Commission of Liaoning, Shenyang, Liaoning, China.

**Ningxia**: Jinbao Ma, Health and Family Planning Commission of Ning Xia Hui Autonomous Region, Yinchuan, Ningxia, China; Juan Hui, Center for Disease Control and Prevention of Ning Xia Hui Autonomous Region, Yinchuan, Ningxia, China; Fu Zhao, Health Supervision Institute of Xixia District in Yinchuan, Ning Xia Hui Autonomous Region, Yinchuan, Ningxia, China.

**Qing Hai**: Jianning Yue, Minru Zhou, Zhihua Xu, Xiaoping Li, Qiongyue Sha, Fuchang Ma, Qing Hai Center for Disease Control and Prevention, Xining, Qinghai, China; Qiuhong Chen, Huiping Bian, Qinghai Cardio-Cerebrovascular Disease Special Hospital, Xining, Qinghai, China.

**Shaanxi**: Jianjun Mu, Tongshuai Guo, Keyu Ren, Chao Chu, First Affiliated Hospital of Xi’an Jiaotong University, Xian, Shaanxi, China.

**Shandong**: Zhendong Liu, Hua Zhang, Yutao Diao, Shangwen Sun, Yingxin Zhao, Institute of Basic Medicine, Shandong Academy of Medical Sciences, Jinan, Shandong, China.

**Shanghai**: Junbo Ge, Jingmin Zhou, Xuejuan Jin, Jun Zhou, Zhongshan Hospital, Fudan University, Shanghai, China.

**Shanxi**: Bao Li, Lijun Zhu, Yuean Zhang, Gang Wang, Shanxi Cardiovascular Hospital, Taiyuan, Shanxi, China; Zhihan Hao, Wuxiang County People's Hospital, Wuxiang, Shanxi, China.

**Sichuan**: Li Cai, Zhou Liu, Zhengping Yong, Jianhong Tao, Yijia Tang, Sichuan Provincial People's Hospital, Chengdu, Sichuan, China; Shaoping Wan, Sichuan Cancer Hospital, Chengdu, Sichuan, China.

**Tianjin**: Zhenshan Jiao, Yuqiang Fan, Tianjin Academy of Traditional Chinese Medicine, Tianjin, China; Hui Gao, Wei Wang, Tianjin Municipal Commission of Health and Family Planning, Tianjin, China; Qingkui Li, Xiaomei Zhou, Tianjin Medical University, Tianjin, China.

**Tibet**: Yundai Chen, Bin Feng, Qinglei Zhu, Sansan Zhou, Chinese People’s Liberation Army General Hospital, Lasha, Tibet, China.

**Xinjiang**: Nanfang Li, Lin Zhou, Delian Zhang, Jing Hong, People's Hospital of Xinjiang Uygur Autonomous Region, Urumuqi, Xinjiang, China.

**Yunnan**: Tao Guo, Min Zhang, First Affiliated Hospital of Kunming Medical University, Kunming, Yunnan, China; Yize Xiao, Center for Disease Prevention and Control of Yunnan, Kunming, Yunnan, China; Xuefeng Guang, Affiliated Yan'an Hospital of Kunming Medical University, Kunming, Yunnan, China.

**Zhejiang**: Xinhua Tang, Jing Yan, Xiaoling Xu, Li Yang, Aimin Jiang, Wei Yu, Zhejiang Hospital, Hangzhou, Zhejiang, China.
